# Mediterranean Dietary Pattern and Risk of Breast Cancer

**DOI:** 10.1371/journal.pone.0055374

**Published:** 2013-02-04

**Authors:** Elisabeth Couto, Sven Sandin, Marie Löf, Giske Ursin, Hans-Olov Adami, Elisabete Weiderpass

**Affiliations:** 1 Department of Medical Epidemiology and Biostatistics, Karolinska Institutet, Stockholm, Sweden; 2 Cancer Registry of Norway, Oslo, Norway; 3 Norwegian Knowledge Centre for the Health Services, Health Economic and Drug Unit, Oslo, Norway; 4 Department of Clinical and Experimental Medicine, University of Linköping, Linköping, Sweden; 5 Department of Biosciences and Nutrition, Karolinska Institutet, Huddinge, Sweden; 6 Department of Nutrition, University of Oslo, Oslo, Norway; 7 Department of Preventive Medicine, University of Southern California, Los Angeles, California, United States of America; 8 Department of Epidemiology, Harvard School of Public Health, Boston, Massachusetts, United States of America; 9 Department of Genetic Epidemiology, Folkhälsan Research Center, Helsinki, Finland; 10 Department of Community Medicine, University of Tromsø, Tromsø, Norway; The University of Texas M. D. Anderson Cancer Center, United States of America

## Abstract

**Background:**

A Mediterranean diet has a recognized beneficial effect on health and longevity, with a protective influence on several cancers. However, its association with breast cancer risk remains unclear.

**Objective:**

We aimed to investigate whether adherence to a Mediterranean dietary pattern influences breast cancer risk.

**Design:**

The Swedish Women’s Lifestyle and Health cohort study includes 49,258 women aged 30 to 49 years at recruitment in 1991–1992. Consumption of foods and beverages was measured at enrollment using a food frequency questionnaire. A Mediterranean diet score was constructed based on the consumption of alcohol, vegetables, fruits, legumes, cereals, fish, the ratio of unsaturated to saturated fat, and dairy and meat products. Relative risks (RR) for breast cancer and specific tumor characteristics (invasiveness, histological type, estrogen/progesterone receptor status, malignancy grade and stage) associated with this score were estimated using Cox regression controlling for potential confounders.

**Results:**

1,278 incident breast cancers were diagnosed. Adherence to a Mediterranean dietary pattern was not statistically significantly associated with reduced risk of breast cancer overall, or with specific breast tumor characteristics. A RR (95% confidence interval) for breast cancer associated with a two-point increment in the Mediterranean diet score was 1.08 (1.00–1.15) in all women, and 1.10 (1.01–1.21) and 1.02 (0.91–1.15) in premenopausal and postmenopausal women, respectively. When alcohol was excluded from the Mediterranean diet score, results became not statistically significant.

**Conclusions:**

Adherence to a Mediterranean dietary pattern did not decrease breast cancer risk in this cohort of relatively young women.

## Introduction

The Mediterranean diet has been inscribed on the heritage list of the United Nations Educational, Scientific and Cultural Organization [1] due, among other things, to its beneficial effects on human health [2]. The key features of the Mediterranean diet are high consumption of vegetables, legumes, fruits, nuts and minimally processed cereals, and mono-unsaturated lipids (coupled with low saturated fat consumption), moderately high consumption of fish, low consumption of dairy and meat products, and regular but moderate intake of alcohol [2]. Adherence to this diet has been reported to reduce overall mortality [3], [4], and to protect against cardiovascular diseases [5], and possibly cancer [6], [7]. However, studies that examined the possible benefit of the Mediterranean diet on specific cancer sites, such as breast cancer, have reported contradictory results [8]–[13].

Hormonal risk factors, such as exposure to endogenous and exogenous hormones [14], are profoundly important in the etiology of breast cancer, and may be more strongly related to the risk of estrogen receptor -positive cancer [15]–[17]. In contrast, non-hormonal risk factors, such as diet, may be more strongly associated with hormone receptor-negative breast cancer. However, few studies have investigated the possible association between a Mediterranean diet and the risk of specific breast tumor characteristics, such as estrogen receptor (ER) and progesterone receptor (PR) status [9], [10]. One study found a protective association with ER-negative breast cancer [10]; another with ER-positive/PR-negative breast cancer [9]. To our knowledge, other specific breast tumor characteristics such as malignancy grade have never been investigated.

Body fatness reduces the risk of premenopausal breast cancer, and increases the risk in postmenopausal women [18]. BMI might also modify the association between Mediterranean diet and breast cancer risk [9], [11]. One study did indeed report a protective association in women with a BMI less than 25 [9], whilst another study found the same association in women with a BMI of 25 or more [11]. One study also reported a protective association among women with lower total energy intake [9].

We used data from the prospective Swedish Women’s Lifestyle and Health (WLH) cohort study to investigate whether adherence to a Mediterranean dietary pattern influences breast cancer risk. We separately examined specific breast cancer tumor characteristics and adjusted for an extensive range of potential confounders.

## Subjects and Methods

### Study Population and Design

As previously described in detail [19], the prospective Swedish WLH cohort study includes women who were aged 30 to 49 years at the time of recruitment in 1991–1992. A total of 96,000 women residing in the Uppsala Health Care Region (including about one-sixth of the Swedish population) were randomly selected from the Swedish Central Population Registry and invited to participate. They were sent an invitation letter and comprehensive questionnaire that requested detailed information on a wide range of factors, including diet, reproductive and hormonal factors, and family history of cancer. Among the 96,000 invited women, 49,258 returned a completed baseline questionnaire.

In 2003, these 49,258 women were sent a follow-up questionnaire, which was completed by 34,402 women. This questionnaire updated information collected in the baseline questionnaire. The WLH study was approved by the Swedish Data Inspection Board and the regional Ethical Committee, Uppsala University, Uppsala, Sweden and the Ethical Committee of the Karolinska Institutet in Stockholm, Sweden. All participants signed an informed consent form.

### Adherence to a Mediterranean Dietary Pattern

The baseline questionnaire included a validated food frequency questionnaire that assessed the frequency and quantity of approximately 80 food items and beverages consumed during the 6 months preceding study recruitment. Reported consumption of foods and beverages were then translated into nutrient and energy intakes using the Swedish National Food Administration database [20].

We measured adherence to a Mediterranean dietary pattern using a variant of the Mediterranean diet score proposed by Trichopoulou et al [2]. The original score considers eight components, selected *a priori*, namely alcohol, vegetables, fruits, legumes, cereals, monounsaturated to saturated fat ratio, dairy and meat products. The variant of the score that we used, also proposed by Trichopoulou et al [3], considers one more component (fish), and measures fat consumption as the ratio of unsaturated to saturated fat to accommodate the low consumption of olive oil-derived monounsaturated lipids in non-Mediterranean countries [21].

We calculated the median consumption of each component in the WLH cohort, and constructed a Mediterranean diet score for each participant based on her consumption of each component compared to that of the overall cohort. We treated components differently according to whether they are traditionally consumed more or less in Mediterranean countries. We assigned components that are more frequently consumed, such as vegetables, fruits and nuts, legumes, cereals, fish and a high ratio of unsaturated to saturated lipids, a value of 1 if a participant’s consumption was above the cohort median for that component, and a value of 0 otherwise. For components that are less frequently consumed in Mediterranean countries (i.e., dairy and meat products) we assigned a value of 1 if a participant’s consumption was below the cohort median, and 0 otherwise.

As alcohol is usually consumed moderately and regularly in Mediterranean countries, we assigned a value of 1 to participants who reported a consumption of 5 to <25 g/day, and a value of 0 for all other intakes. We then summed the values for all components (equal to either 0 or 1) to obtain a participant’s Mediterranean diet score. The score varied between 0 and 9; the higher the score, the closer the adherence to a Mediterranean dietary pattern. Because alcohol is an established risk factor for breast cancer [22], we also analyzed the Mediterranean diet score without the alcohol component and controlled for alcohol as a confounder in the statistical model. This was done when examining breast cancer risk overall, and the risk of specific breast tumor characteristics.

### Follow-up and Endpoints

All participants were followed up through linkages with nationwide health registers using the individually unique national registration number assigned to all legal residents in Sweden. The study population was linked to the population register for information on emigration and death. Data on breast cancer was obtained through linkage with the national cancer register through 2008, and the breast cancer quality register in the Uppsala health care region (that started in September 1992). Information on breast tumor characteristics was available in the regional, but not in the national cancer register. During follow-up, 322 participants had a diagnosis of breast cancer (International Classification of Diseases, 7th revision, code 170.0) in the national cancer register that was not recorded in the regional quality breast cancer register in Uppsala, as they had moved out of Uppsala County after cohort recruitment.

### Menopausal Status

Information from both baseline and follow-up questionnaires was used to determine menopausal status. We extracted data on oophorectomy and hysterectomy from the national in-patient register [23]. We used this information to verify surgeries reported by participants in the baseline questionnaire, and to update information on surgical menopause during follow-up. A woman was defined as postmenopausal if her menstruation had stopped, or if both ovaries had been surgically removed. We classified participants who did not provide any information on menstrual status, who reported not menstruating but did not give the reason, who had had a hysterectomy, or had bleeding due to use of hormone therapy or intra-uterine device as having unknown menopausal status. However, we considered any participants with unknown menopausal status as postmenopausal when they reached the age when natural menopause occurred in 90% of the cohort, i.e., 54 years for ever-smokers; 55 for never-smokers and participants with unknown smoking history [24]. We applied this same age rule to postmenopausal participants with missing information on age at start of menopause.

### Statistical Analyses

Participants were excluded if they had been diagnosed with breast cancer before or at recruitment (N = 244). In addition, 981 participants with a total energy intake outside the 1^st^ and 99^th^ percentiles (1,846.6 and 12,473.9 kJ/day, respectively) were excluded.

Crude distributions of various possible breast cancer risk factors were calculated, and we examined how these factors were associated with breast cancer risk in the WLH study by estimating RRs for breast cancer in premenopausal and postmenopausal women.

The association between breast cancer incidence and adherence to a Mediterranean dietary pattern was examined by fitting Cox regression with attained age as a time scale, considering all participants together, as well as premenopausal and postmenopausal participants separately. We followed up premenopausal participants from the date the baseline questionnaire was completed until primary breast cancer diagnosis, death, emigration, end of follow-up (31 December 2008), or the date of menopause, whichever occurred first. For postmenopausal participants, women-years were calculated from the date of menopause or the date the baseline questionnaire was completed (whichever occurred last), until the date of primary breast cancer diagnosis, death, emigration, or 31 December 2008 (whichever occurred first). Therefore, participants who were both premenopausal and postmenopausal during follow-up are included in the stratified analyses of both premenopausal and postmenopausal participants.

As information on specific breast tumor characteristics, obtained through the regional quality breast cancer register in Uppsala, was available as from September 1992, we used this date as the start of follow-up when examining these characteristics. When examining a specific breast tumor characteristic, the event of interest was that characteristic, and participants with other breast tumor characteristics or with missing information were censored at breast cancer diagnosis.

Relative risks (RR) and corresponding two-sided 95% Wald type CIs were estimated by calculating hazard ratios. The Cox proportional hazards assumption was checked assessing graphs of scaled Schoenfeld residuals *versus* time [25]. Hazard ratios were calculated for both a two-point increment in the Mediterranean diet score (treated as a linear continuous covariate), and for categories of score (0–2, 3–4, 5, 6–7, 8–9). Unless otherwise stated, we adjusted for the following baseline characteristics: history of breast cancer in mother and/or sister(s) (categorical, no/yes), personal history of benign breast disease (categorical, no/yes), smoking status (categorical, never/former/current), BMI (continuous, kg/m^2^), height (continuous, cm), age at first birth and total number of children (categorical, no children; age at first birth <30 and 1 child, 2 children, and ≥3 children; and age at first birth ≥30, and 1 child, 2 children, and ≥3 children), educational level (continuous, years), age at menarche (continuous, years), total energy intake (continuous, kJ/day), consumption of beverages (continuous, grams/day), potatoes (continuous, grams/day), sweets (continuous, grams/day), and eggs (continuous, grams/day). These possible confounding factors were selected *a priori* due to their possible effect on breast cancer risk and their potential association with diet. Potatoes intake was consider as a possible confounder because while being a vegetable, the effect of potatoes consumption on health has been reported to differ from the commonly acknowledged beneficial effect of vegetables. Meat consumption is one the component of the Mediterranean diet score and we adjusted the analyses for eggs intake as it is another high source of animal protein. Our analyses were adjusted for energy intake, sweets, whose consumption has been reported to be detrimental for health, are a high source of energy and we, therefore, adjusted analyses for sweets consumption. We performed the statistical analyses adjusting also for physical activity and found similar results. However, due to lack of completeness of physical activity reporting (2392 missing answers), this variable was not included in the final model. Possible modifying effects of BMI, age at menarche, age at first birth and number of children, history of breast cancer in mother and/or sister(s), smoking status, total energy intake at baseline, and ever-use of oral contraceptive and hormone therapy at follow-up were ascertained by adding interaction terms to the statistical model. We used baseline and follow-up questionnaires to ascertain hormone therapy and oral contraceptive use: women who reported to have never used these in both questionnaires were considered never users. If a woman reported using hormone therapy and oral contraceptives at baseline or follow-up, she was classified as an ever user, and women who did not complete the follow-up questionnaire, or did not reply to this question in both questionnaires were classified in the “unknown” category. Analyses were further adjusted for use of oral contraceptives and hormone therapy (categorical, never/ever/unknown). For overall analyses, menopausal status was entered in the model as a time-dependent variable (in addition to the above-mentioned covariates). We excluded 3,193 participants with missing information on at least one of the following covariates: BMI (N = 1,802), height (N = 904), age at first birth and total number of children (N = 3), age at menarche (N = 579), or use of oral contraceptives (N = 49).

We used Cox regression to examine premenopausal and postmenopausal participants separately. We estimated RRs for breast cancer associated with each component of the Mediterranean diet score. These analyses were mutually adjusted for other score’s components, further to the adjustments mentioned above. We also examined the association between Mediterranean dietary pattern and breast cancer risk among non-alcohol drinkers only for breast cancer overall, as well as for specific breast tumor characteristics. All analyses were performed using the statistical software SAS version 9.2 (SAS Institute Inc., Cary, North Carolina, USA).

In this report, we examined *in situ* and invasive breast cancers together. We also carried out all analyses presented here for *in situ* and invasive tumors separately, and found similar results (results not shown). For this, we used data from the regional quality breast cancer register (that gave information on the invasiveness of the tumor), and considered 1,176 invasive and 188 *in situ* breast cancers (in their lifetime, women could have been diagnosed with both invasive and *in situ* breast cancers).

## Results

The 44,840 participants included in the final analyses were followed up for 16 years on average, during which 1,278 incident breast cancers were diagnosed. Having three or more children, especially below age 30 years, was associated with a decrease in breast cancer risk in premenopausal women (Table 1). Furthermore, participants with a mother and/or sister(s) diagnosed with breast cancer had a RR for breast cancer of 1.90 (95% CI: 1.46–2.46) in premenopausal women, and 1.61 (95% CI: 1.16–2.23) in postmenopausal women compared to participants without such a family history.

**Table 1:** Characteristics of the study participants and associated RRs and corresponding 95% CIs for breast cancer by menopausal status.

		Premenopausal					Postmenopausal		
Characteristics		*n* (%)		Breast cancer cases *n* (%)		RR (95% CI)^1^	*n* (%)	Breast cancercases *n* (%)	RR (95% CI)^1^
**Overall**		40031 (100)		736 (100)			27509 (100)	448 (100)	
**BMI at baseline (kg/m** 2 **)**	≤20	5032 (12.6)		105 (14.3)		1.00	2869 (10.4)	36 (8.0)	1.00
	21–24	24959 (62.3)		459 (62.4)		0.90 (0.72–1.11)	16892 (61.4)	276 (61.6)	1.18 (0.83–1.68)
	≥25	10040 (25.1)		172 (23.4)		0.88 (0.68–1.12)	7748 (28.2)	136 (30.4)	1.24 (0.85–1.80)
**Age at menarche (years)**	≤12	14870 (37.2)		253 (34.4)		1.00	9726 (35.4)	159 (35.5)	1.00
	13	12118 (30.3)		238 (32.3)		1.13 (0.95–1.35)	8112 (29.5)	136 (30.4)	0.96 (0.76–1.21)
	≥14	13043 (32.5)		245 (33.3)		1.07 (0.89–1.27)	9671 (35.2)	153 (34.1)	0.86 (0.69–1.08)
**Age at first birth (years), total number of children at baseline**	No children	5539 (13.8)		132 (17.9)		1.00	3041 (11.0)	49 (10.9)	1.00
	<30, 1 child	3615 (9.0)		74 (10.0)		0.84 (0.63–1.12)	2630 (9.6)	45 (10.0)	1.00 (0.67–1.50)
	<30, 2 children	1563 (36.4)		265 (36.0)		0.74 (0.60–0.91)	10529 (38.3)	176 (37.3)	0.90 (0.65–1.24)
	<30, ≥3 children	10297 (25.7)		151 (20.5)		0.58 (0.46–0.73)	7633 (27.7)	118 (26.4)	0.92 (0.66–1.28)
	≥30, 1 child	2519 (6.3)		43 (5.8)		0.69 (0.49–0.98)	1280 (4.6)	31 (6.9)	1.37 (0.87–2.15)
	≥30, 2 children	2789 (6.9)		60 (8.1)		0.80 (0.59–1.09)	1825 (6.6)	28 (6.2)	0.95 (0.60–1.52)
	≥30, ≥3 children	715 (1.8)		11 (1.5)		0.52 (0.28–0.97)	571 (2.1)	10 (2.2)	1.20 (0.61–2.38)
**Ever oral contraceptive use at follow-up**	Never	5868 (14.7)		100 (13.6)		1.00	2821 (10.2)	45 (10.0)	1.00
	Ever	24274 (60.6)		441 (59.9)		1.00 (0.80–1.25)	18453 (67.1)	307 (68.5)	0.76 (0.55–1.04)
	Unknown^2^	9889 (24.7)		195 (26.5)		1.07 (0.72–1.58)	6235 (22.7)	96 (21.4)	0.86 (0.53–1.39)
**Ever hormone therapy use at follow-up**	Never	…		…		…	9523 (34.6)	118 (26.3)	1.00
	Ever	…		…		…	10603 (38.5)	223 (49.8)	1.21 (0.96–1.51)
	Unknown^3^	…		…		…	7383 (26.8)	107 (23.9)	1.04 (0.70–1.55)
**History of breast cancer in mother and/or sister(s) at baseline**	No		38215 (95.5)		674 (91.6)	1.00	26062 (94.7)	408 (91.1)	1.00
	Yes		1816 (4.5)		62 (8.4)	1.90 (1.46–2.46)	1447 (5.3)	40 (8.9)	1.61 (1.16–2.23)
**Smoking status at baseline**	Never		16700 (41.7)		301 (40.9)	1.00	10908 (39.6)	160 (35.7)	1.00
	Former		14927 (37.3)		277 (37.6)	1.01 (0.85–1.18)	10309 (37.5)	187 (41.7)	1.28 (1.03–1.58)
	Current		8404 (21.0)		158 (21.5)	1.03 (0.85–1.26)	6292 (22.9)	101 (22.5)	1.26 (0.98–1.62)
**Total energy intake at baseline (in kJ)**	<5590		12652 (31.6)		233 (31.7)	1.00	10279 (37.4)	170 (37.9)	1.00
	5590−<7725		13787 (34.4)		272 (37.0)	1.08 (0.90–1.29)	9434 (34.3)	160 (35.7)	1.09 (0.87–1.35)
	≥7225		13592 (33.9)		231 (31.4)	0.94 (0.78–1.13)	7796 (28.3)	118 (26.3)	1.02 (0.80–1.29)

1 Analyses were adjusted for all characteristics presented in the table, as well as height, educational level, alcohol consumption, and personal history of benign disease.

2 Includes women who reported no oral contraceptive use at baseline and did not complete the follow-up questionnaire.

3 Includes women who reported no hormone therapy use at baseline and did not complete the follow-up questionnaire.

**Abbreviations:** n, number of participants, RR, relative risk.

Participants with a higher Mediterranean diet score had a lower BMI, were more likely to have a mother and/or a sister(s) with breast cancer, to be never- or former- smokers, to have used oral contraceptives and/or hormone therapy, and to have a moderate alcohol consumption *versus* a lower consumption, compared with participants with a lower Mediterranean diet score (Table 2). Participants with a score of 8–9 had a higher consumption of all components of the score except dairy and meat products, compared to participants with a score of 0–2, and a particularly higher consumption of alcohol, vegetables, fruits, fish, and legumes.

**Table 2:** Participant characteristics by categories of the Mediterranean diet score.

		Categories of the Mediterranean diet score^1^									
		0–2		3–4		5		6–7		8–9	
	Characteristics	*N*	(%)	*n*	(%)	*n*	(%)	*n*	(%)	*n*	(%)
BMI at baseline (in kg/m^2^)	≤20	1062	(14.1)	2244	(12.3)	1066	(11.7)	1060	(11.5)	116	(15.3)
	21–24	4493	(59.5)	11240	(61.6)	5675	(62.4)	5875	(63.9)	494	(65.0)
	≥25	1995	(26.4)	4759	(26.1)	2356	(25.9)	2255	(25.9)	150	(19.7)
Age at menarche (years)	≤12	2776	(36.8)	6840	(37.5)	3432	(37.7)	3434	(37.4)	291	(38.3)
	13	2266	(30.0)	5490	(30.1)	2768	(30.4)	2791	(30.4)	199	(26.2)
	≥14	2508	(33.2)	5913	(32.4)	2897	(31.8)	2965	(32.3)	270	(35.5)
Age at first birth (years) and total number of children at baseline	No children	994	(13.2)	2512	(13.8)	1273	(14.0)	1313	(14.3)	130	(17.1)
	<30, 1 child	757	(10.0)	1722	(9.4)	787	(8.6)	831	(9.0)	83	(10.9)
	<30, 2 children	2735	(36.2)	6700	(36.7)	3311	(36.4)	3382	(36.8)	289	(38.0)
	<30, ≥3 children	2049	(27.1)	4677	(25.6)	2345	(25.8)	2305	(25.1)	158	(20.8)
	≥30, 1 child	449	(5.9)	1114	(6.1)	582	(6.4)	551	(6.0)	36	(4.7)
	≥30, 2 children	463	(6.1)	1220	(6.7)	636	(7.0)	635	(6.9)	52	(6.8)
	≥30, ≥3 children	103	(1.4)	298	(1.6)	163	(1.8)	173	(1.9)	12	(1.6)
Ever oral contraceptive use at follow-up	Never	1228	(22.2)	2812	(20.6)	1298	(18.5)	1254	(17.5)	98	(16.4)
	Ever	4310	(77.8)	10860	(79.4)	5713	(81.5)	5923	(82.5)	498	(83.6)
Ever hormone therapy use at follow-up	Never	3384	(64.4)	7882	(61.1)	3967	(59.5)	3962	(58.1)	307	(53.4)
	Ever	1868	(35.6)	5018	(38.9)	2699	(40.5)	2853	(41.9)	268	(46.6)
History of breast cancer in mother and/or sister(s) at baseline	No	7242	(95.9)	17427	(95.5)	8687	(95.5)	8731	(95.0)	709	(93.3)
	Yes	308	(4.1)	816	(4.5)	410	(4.5)	459	(5.0)	51	(6.7)
Smoking status at baseline	Never	2791	(37.0)	7371	(40.4)	3972	(43.7)	4015	(43.7)	305	(40.1)
	Former	2711	(35.9)	6796	(37.2)	3377	(37.1)	3558	(38.7)	338	(44.5)
	Current	2048	(27.1)	4076	(22.3)	1748	(19.2)	1617	(17.6)	117	(15.4)
Alcohol consumption at baseline (in g/day)	<5	6985	(92.5)	15247	(83.6)	7157	(78.7)	6258	(68.1)	277	(36.5)
	5–25	521	(6.9)	2919	(16.0)	1917	(21.1)	2910	(31.7)	483	(63.5)
	≥25	44	(0.6)	77	(0.4)	23	(0.2)	22	(0.2)	0	(0)

				Categories of the Mediterranean diet score^1^									
		All scores (0–9)		0–2		3–4		5		6–7		8–9	
		Mean	(SD)	Mean	(SD)	Mean	(SD)	Mean	(SD)	Mean	(SD)	Mean	(SD)
**Nutritional variables at baseline**	Alcohol consumption (g/day)	3.0	(4.2)	2.1	(4.1)	2.8	(4.3)	3.1	(3.9)	3.8	(4.2)	5.7	(4.1)
	Alcohol consumption (% energy^2^)	1.4	(2.0)	1.1	(1.8)	1.4	(2.1)	1.5	(1.9)	1.7	(1.9)	2.4	(1.7)
	Vegetables (g/day)	70.4	(49.5)	38.3	(27.4)	60.9	(40.9)	83.9	(50.5)	98.5	(55.6)	113.2	(55.0)
	Fruits (g/day)	158.1	(118.1)	88.8	(76.4)	140.7	(107.4)	183.9	(117.2)	216.2	(125.9)	252.4	(137.2)
	Legumes (g/day)	19.3	(19.6)	9.1	(11.6)	15.6	(16.6)	23.2	(19.6)	29.9	(23.1)	36.2	(21.2)
	Cereals (g/day)	195.6	(85.4)	162.1	(71.7)	188.1	(83.6)	206.4	(86.1)	223.5	(86.9)	242.0	(77.0)
	Fish (g/day)	24.4	(15.7)	14.9	(9.4)	21.7	(13.5)	28.0	(16.2)	33.0	(16.9)	38.8	(19.2)
	Unsaturated/saturated fat (g/day)	1.1	(0.2)	0.9	(0.1)	1.1	(0.2)	1.1	(0.2)	1.2	(0.2)	1.3	(0.2)
	Dairy (g/day)	369.8	(284.1)	501.8	(293.5)	387.1	(289.6)	340.8	(269.0)	272.3	(234.0)	168.5	(138.6)
	Meat (g/day)	87.7	(41.4)	84.6	(39.7)	87.5	(40.2)	90.6	(42.2)	89.0	(44.1)	74.7	(38.9)
	Total energy	6525.2	(1880.4)	6035.1	(1807.5)	6377.3	(1907.9)	6756.0	(1867.0)	6951.3	(1789.0)	7026.0	(1546.2)
	Total fat (% energy^2^)	30.7	(5.4)	33.3	(5.4)	31.2	(5.3)	30.0	(5.1)	28.6	(5.1)	26.8	(5.1)
	Carbohydrates (% energy^2^)	51.1	(6.0)	48.1	(5.5)	50.6	(5.8)	52.1	(5.7)	53.6	(5.9)	55.6	(5.7)
	Proteins (% energy^2^)	16.5	(2.5)	17.4	(2.5)	16.6	(2.5)	16.3	(2.4)	15.8	(2.2)	14.9	(1.8)

1 The Mediterranean score includes the consumption of the following components: alcohol, vegetables, fruits, legumes, cereals, fish, unsaturated to saturated fat ratio, and dairy and meat products. Its value ranges from 0 to 9 with a high value corresponding to a high adherence to the Mediterranean dietary pattern as defined in the present report.

2 Percent energy of total energy intake.

**Abbreviations:** n, number of participants.

The possible association between Mediterranean dietary pattern was examined separately in all women and in premenopausal or postmenopausal women (Table 3). Adhering to such a diet was not associated with a decrease in breast cancer risk in these subgroups. The RRs for breast cancer associated with a two-point increment in the Mediterranean diet score was 1.08 (95% CI: 1.00–1.15) for all women, 1.10 (95% CI: 1.01–1.21) in premenopausal, and 1.02 (95% CI: 0.91–1.15) in postmenopausal participants. Corresponding unadjusted RRs were 1.08 (95% CI: 1.01–1.16), 1.10 (95% CI: 1.00–1.20), and 1.05 (0.94–1.17), respectively (data not shown). When we excluded alcohol from the score, and adjusted for alcohol consumption, the RR for all women was 1.06 (95% CI: 0.98–1.13). The corresponding RRs were 1.07 (95% CI: 0.98–1.18) for premenopausal and 1.02 (95% CI: 0.90–1.15) for postmenopausal women. The RRs for breast cancer associated with a two-point increment in the Mediterranean diet score for non-alcohol drinkers were 1.03 (95% CI: 0.87–1.23) overall, 0.94 (95% CI: 0.75–1.17) in premenopausal, and 1.17 (95% CI: 0.86–1.59) in postmenopausal participants (data not shown). No statistically significant interaction was found between Mediterranean diet score and BMI (p for interaction = 0.17), age at menarche (p = 0.14), age at first birth and number of children (p = 0.44), history of breast cancer in mother and/or sister(s) (p = 0.99), smoking status (p = 0.66), total energy intake at baseline (p = 0.55), and ever-use of oral contraceptive (p = 0.23) and hormone therapy (p = 0.58) at follow-up (results not shown).

**Table 3:** RR and 95% CI for breast cancer according to categories or a two-point increment in the Mediterranean diet score.

	Full Mediterranean diet score^1^	Cases (*n*)	RR (95% CI)^2^	Mediterranean diet score excluding the alcohol component^3^	Cases (*n*)	RR (95% CI)^4^
**All women**	0–2	196	1.00	0–2	226	1.00
	3–4	487	0.98 (0.83–1.16)	3	235	0.98 (0.82–1.18)
	5	263	1.03 (0.85–1.23)	4–5	561	1.07 (0.93–1.25)
	6–7	297	1.10 (0.92–1.33)	6–7	221	1.07 (0.88–1.29)
	8–9	35	1.42 (0.99–2.05)	8	35	1.42 (0.99–2.03)
			Ptrend = 0.05			Ptrend = 0.12
	Two-point increment in the score	1278	1.08 (1.00–1.15)	Two-point increment in the score	1278	1.06 (0.98–1.13)
**Premenopausal women** 5	0–2	117	1.00	0–2	131	1.00
	3–4	284	0.98 (0.79–1.22)	3	135	0.98 (0.77–1.25)
	5	155	1.07 (0.84–1.37)	4–5	328	1.13 (0.92–1.39)
	6–7	153	1.04 (0.81–1.33)	6–7	115	1.03 (0.79–1.33)
	8–9	27	2.12 (1.39–3.24)	8	27	2.17 (1.42–3.30)
			Ptrend = 0.08			Ptrend = 0.05
	Two-point increment in the score	736	1.10 (1.01–1.21)	Two-point increment in the score	736	1.07 (0.98–1.18)
**Postmenopausal women** 5	0–2	66	1.00	0–2	82	1.00
	3–4	166	0.93 (0.69–1.23)	3	79	0.89 (0.65–1.21)
	5	84	0.85 (0.61–1.17)	4–5	186	0.89 (0.68–1.15)
	6–7	125	1.14 (0.84–1.55)	6–7	94	1.08 (0.79–1.46)
	8–9	7	0.63 (0.29–1.37)	8	7	0.59 (0.27–1.28)
			Ptrend = 0.55			Ptrend = 0.89
	Two-point increment in the score	448	1.02 (0.91–1.15)	Two-point increment in the score	448	1.02 (0.90–1.15)

1 The Mediterranean score includes the consumption of the following components: alcohol, vegetables, fruits, legumes, cereals, fish, unsaturated to saturated fat ratio, and dairy and meat products. Its value ranges from 0 to 9 with a high value corresponding to a high adherence to the Mediterranean dietary pattern as defined in the present report.

2 Analyses were adjusted for history of breast cancer in mother and/or sister(s), personal history of benign breast disease, smoking status, BMI, height, age at first birth and number of children, educational level, age at menarche, total energy intake, consumption of beverages, potatoes, sweets, and eggs.

3 This score excludes the alcohol component and considers only the eight remaining components listed in *^1^*. Its value ranges from 0 to 8.

4 Further to the variables listed in *^2^*, analyses were also adjusted for alcohol consumption.

5 The same woman can be in both premenopausal and postmenopausal categories, if they were both premenopausal and postmenopausal during the follow-up period.

**Abbreviations:** n, number of participants, RR, relative risk.

When examining the possible association between the nine components’ of the Mediterranean diet score and breast cancer risk in all women and in premenopausal and postmenopausal women (Table 4), we found a statistically significant reduction in breast cancer risk with dairy products consumption. The RRs for breast cancer associated with an increment in daily intake of dairy products of 290 g were 0.93 (0.87–0.99), 0.93 (0.86–0.99), and 0.89 (0.80–0.98) in all women, and premenopausal and postmenopausal women, respectively. No statistically significant association was observed for other components.

**Table 4:** RR and 95% CI for breast cancer associated with increments in the components of the Mediterranean diet score.

		All women		Premenopausal women		Postmenopausal women	
Components	Increment^1^	Mean (SD) consumption	RR (95% CI)^2^	Mean (SD) consumption	RR (95% CI) 2	Mean (SD) consumption	RR (95% CI) 2
Alcohol (g/day)	5	3.0 (4.2)	1.04 (0.99–1.09)	3.0 (4.2)	1.03 (0.98–1.09)	3.2 (4.4)	1.05 (0.98–1.13)
Cereals (g/day)	85	195.6 (85.4)	1.02 (0.95–1.10)	197.2 (85.2)	1.00 (0.92–1.09)	187.6 (84.2)	1.04 (0.92–1.17)
Dairy products (g/day)	290	369.8 (284.1)	0.93 (0.87–0.99)	371.8 (283.1)	0.93 (0.86–0.99)	353.4 (278.6)	0.89 (0.80–0.98)
Fish (g/day)	15	24.4 (15.7)	0.98 (0.93–1.04)	24.4 (15.6)	1.00 (0.94–1.05)	24.9 (16.0)	1.01 (0.93–1.09)
Fruits and nuts (g/day)	120	158.1 (118.1)	0.95 (0.89–1.00)	158.5 (117.6)	0.95 (0.89–1.01)	159.1 (119.9)	0.97 (0.89–1.06)
Legumes (g/day)	20	19.3 (19.6)	0.94 (0.89–1.00)	19.3 (19.5)	0.94 (0.89–1.00)	19.3 (19.2 )	0.96 (0.87–1.04)
Meat products (g/day)	40	87.7 (41.1)	0.99 (0.93–1.05)	88.2 (41.5)	0.96 (0.90–1.03)	84.1 (39.8)	1.04 (0.94–1.15)
Vegetables (g/day)	50	70.4 (49.5)	1.01 (0.95-1.07)	70.3 (49.0)	1.02 (0.96–1.08)	71.6 (49.4)	0.97 (0.89–1.06)
Ratio of unsaturated to saturated lipids	1	1.1 (0.2)	0.91 (0.69–1.20)	1.1 (0.2)	1.01 (0.75–1.36)		0.66 (0.43–1.02)

1 The increment is approximately equal to the component’s standard deviation.

2 Analyses were adjusted for history of breast cancer in mother and/or sister(s), personal history of benign breast disease, smoking status, BMI, height, age at first birth and number of children, educational level, age at menarche, total energy intake, consumption of beverages, potatoes, sweets, eggs; further to be mutually adjusted for the scores components listed in the table.

**Abbreviations:** SD, standard deviation, g, grams, RR, relative risk.

We investigated the risk of several breast cancer tumor characteristics associated with adherence to a Mediterranean dietary pattern (Table 5). No decrease in the risk of examined breast tumor characteristics (i.e., invasiveness, histological type, ER and PR status, malignancy grade and stage) was found overall, or in premenopausal and postmenopausal participants. The RRs for ER-negative breast cancer associated with a two-point increment in the Mediterranean diet score were 1.19 (95% CI: 0.96–1.47) in premenopausal, and 1.17 (95% CI: 0.87–1.59) in postmenopausal participants. For ER-positive breast cancer, the corresponding RRs were 1.02 (95% CI: 0.91–1.14), and 0.98 (95% CI: 0.86–1.11). The RRs for ER- and PR-negative breast cancer were 1.34 (95% CI: 1.05–1.71) in premenopausal, and 1.18 (95% CI: 0.86–1.62) in postmenopausal participants (Table 5). After excluding alcohol consumption from the Mediterranean diet score, and controlling the analyses for it, corresponding RRs were 1.28 (95% CI: 1.00–1.65) and 1.14 (95% CI: 0.82–1.58), respectively (data not shown). We also examined the association between adherence to a Mediterranean dietary pattern and the risk of ER- and PR-negative tumors among non-alcohol drinkers only. The resultant RRs were 0.83 (95% CI: 0.40–1.73), 1.88 (95% CI: 0.93–3.79), and 1.13 (95% CI: 0.74–1.72), in premenopausal, postmenopausal and overall, respectively (data not shown).

**Table 5:** RR and 95% CI for specific breast tumor characteristics associated with a two-point increment in the Mediterranean diet score.

			Premenopausal^1^			Postmenopausal^1^				All women					
			Cases (*n*)	Mean score^2^ (SD)	RR (95% CI)^3^	Cases (*n*)	Mean score^2^ (SD)	RR (95% CI)^3^		Cases (*n*)	Mean score^2^ (SD)		RR (95% CI)^3^		
**Invasiveness of the tumor**	Invasive		666	4.31 (1.72)	1.10 (1.00–1.21)	421	4.37 (1.73)		1.02 (0.90–1.14)	1176		4.33 (1.69)		1.07 (0.99–1.15)	
	*In situ*		126	4.40 (1.72)	1.13 (0.91–1.40)	51	4.49 (1.67)		1.17 (0.83–1.65)	188		4.40 (1.68)		1.14 (0.95–1.36)	
**Histological type**	Ductal		560	4.33 (1.75)	1.10 (0.99–1.22)	365	4.32 (1.74)		0.98 (0.86–1.12)	1006		4.31 (1.70)		1.05 (0.97–1.13)	
	Lobular		91	4.04 (1.65)	0.92 (0.71–1.19)	58	4.44 (1.75)		1.37 (1.00–1.89)	157		4.31 (1.71)		1.08 (0.89–1.31)	
	Medullar		14	4.71 (1.64)	1.57 (0.82–3.01)	3	4.58 (1.44)		0.91 (0.22–3.79)	19		4.42 (1.54)		1.32 (0.75–2.30)	
**ER status**	Positive		478	4.24 (1.71)	1.02 (0.91–1.14)	347	4.32 (1.73)		0.98 (0.86–1.11)	874		4.29 (1.69)		1.01 (0.93–1.10)	
	Negative		131	4.35 (1.56)	1.19 (0.96–1.47)	64	4.40 (1.63)		1.17 (0.87–1.59)	227		4.34 (1.61)		1.14 (0.97–1.34)	
**PR status**	Positive		425	4.24 (1.70)	1.02 (0.91–1.15)	250	4.30 (1.72)		0.98 (0.84–1.15)	722		4.28 (1.68)		1.01 (0.92–1.11)	
	Negative		177	4.35 (1.56)	1.15 (0.95–1.38)	158	4.43 (1.69)		1.05 (0.87–1.28)	368		4.36 (1.67)		1.09 (0.96–1.24)	
**ER/PR status**	ER+/PR+		395	4.28 (1.70)	1.05 (0.93–1.18)	246	4.34 (1.73)		0.97 (0.83–1.14)	680		4.30 (1.68)		1.03 (0.93–1.13)	
	ER−/PR–		102	4.50 (1.54)	1.34 (1.05–1.71)	59	4.57 (1.63)		1.18 (0.86–1.62)	185		4.45 (1.60)		1.23 (1.03–1.47)	
	ER+/PR–		75	4.15 (1.78)	0.93 (0.70–1.23)	99	4.29 (1.74)		0.98 (0.77–1.25)	183		4.26 (1.73)		0.98 (0.81–1.17)	
	ER−/PR+		30	3.77 (1.57)	0.77 (0.49–1.20)	4	3.74 (1.50)		3.07 (0.60–15.75)	42		3.86 (1.57)		0.82 (0.56–1.20)	
**Malignancy grade**	Grade 1	138		4.12 (1.71)	0.92 (0.75–1.14)	121	4.16 (1.65)		0.82 (0.66–1.03)	268		4.15 (1.66)		0.88 (0.76–1.02)	
	Grade 2	223		4.30 (1.81)	1.07 (0.90–1.25)	190	4.45 (1.80)		1.16 (0.97–1.38)	443		4.40 (1.73)		1.12 (0.99–1.25)	
	Grade 3	132		4.35 (1.65)	1.13 (0.91–1.39)	104	4.40 (1.75)		1.08 (0.85–1.37)	256		4.35 (1.69)		1.08 (0.93–1.26)	
**Stage**	I	220		4.31 (1.71)	1.06 (0.90–1.25)	203	4.28 (1.68)		0.92 (0.78–1.09)	451		4.29 (1.69)		1.00 (0.89–1.12)	
	II	245		4.23 (1.70)	1.03 (0.88–1.20)	145	4.37 (1.80)		1.11 (0.91–1.37)	421		4.31 (1.69)		1.05 (0.93–1.18)	
	III and IV	22		4.41 (2.26)	1.23 (0.73–2.07)	21	4.61 (1.73)		1.11 (0.65–1.89)	46		4.39 (1.88)		1.17 (0.82–1.68)	

1 The same woman can be in both premenopausal and postmenopausal categories, if they were both premenopausal and postmenopausal during the follow-up period.

2 The Mediterranean score includes the consumption of the following components: alcohol, vegetables, fruits, legumes, cereals, fish, unsaturated to saturated fat ratio, and dairy and meat products. Its value ranges from 0 to 9 with a high value corresponding to a high adherence to the Mediterranean dietary pattern as defined in the present report.

3 Analyses were adjusted for history of breast cancer in mother and/or sister(s), personal history of benign breast disease, smoking status, BMI, height, age at first birth and number of children, educational level, age at menarche, total energy intake, consumption of beverages, potatoes, sweets, and eggs.

**Abbreviations:** ER, estrogen receptor; n, number of participants, PR, progesterone receptor; RR, relative risk.

## Discussion

Adherence to a Mediterranean dietary pattern was not associated with reduced risk of breast cancer overall, nor of specific breast tumor characteristics, overall, or in premenopausal and postmenopausal participants. Previous studies investigating the possible association between a Mediterranean diet and breast cancer risk have reported conflicting results. While some studies found no association of this diet with the risk of breast cancer overall [8], in premenopausal [11], [12], or in postmenopausal women [10], [11], a protective association in postmenopausal women [9], [12], Hispanic postmenopausal women [11], and Asian American postmenopausal women [13] has been described. Indeed, studies that found a protective association of a Mediterranean diet with breast cancer risk have reported this association in postmenopausal women only [9], [11]–[13]. While WLH study participants may have reached menopause during follow-up, they are somewhat younger (30 to 49 years in 1991–1992) than populations in many other studies, and it is possible that results may differ in older women.

The Greek EPIC cohort found a reduction in total mortality with closer adherence to a Mediterranean diet, and reported that moderate alcohol consumption was one of the components with greater contribution to this association [26]. Moderate alcohol consumption, which is a risk factor for breast cancer [27], increased our score, contrary to lower or higher consumption. However, when we excluded alcohol consumption from the score, no statistically significant association was found. One published study treated alcohol as a negative factor, (giving a value of 1 to consumptions above the median, and a value of 0 otherwise), and found a protective association [13].

Risk factors for breast cancer might differ by hormone receptor status [28]. Previous studies have examined whether a Mediterranean diet has a different influence on the risk of ER- and PR-negative and positive breast cancer, and have reported conflicting results [9], [10]. The Nurses Health Study cohort found no association with ER-positive breast cancer risk, and an inverse association with ER-negative breast cancer [10]. However, data from the French EPIC cohort showed no association of a Mediterranean diet with ER-negative breast cancer, and a protective association with ER-positive/PR-negative tumors [9]. We found no association between adherence to a Mediterranean dietary pattern and the risk of ER- or PR-negative cancer. When we examined ER and PR receptor statuses jointly, we found an increased risk of ER-negative/PR-negative tumors both overall and in premenopausal women. However, these results might be due to chance, and were not statistically significant when we excluded alcohol from the Mediterranean diet score. We also examined the possible association between adherence to a Mediterranean dietary pattern and risk of ER-negative/PR-negative tumors among non-alcohol drinkers, and found no association overall, in premenopausal, or in postmenopausal participants.

Previous studies have used different methods to ascertain adherence to a Mediterranean diet. Most of them used pattern identification methods to define a specific dietary pattern [9], [29]. A limitation of this method is that the identified pattern is population-dependent, and may not apply to other populations. Other studies have used a method similar to ours, constructing a score assuming an *a priori* knowledge of the composition of the Mediterranean diet [8], [12]. Both methods ascertain dietary patterns, which measure the complexity of diet more accurately than simply considering individual food items [30]. A limitation of the Mediterranean diet score is that the composition of a specific component might vary from one population to another. For example, in Mediterranean countries olive oil is the main source of unsaturated fat, unlike in non-Mediterranean countries. However, the ratio of unsaturated to saturated fat used in the present report accommodates the low consumption of olive oil-derived monounsaturated lipids in non-Mediterranean countries. Differences in the methods used to ascertain Mediterranean diet may explain the discrepancies in the results of published studies.

The strengths of the WLH study are its prospective design, and extensive information on potential confounders. Furthermore, the use of national registries to identify disease outcome allowed for an almost complete follow-up. Diet was measured using a validated food frequency questionnaire [31], and it has been shown that these questionnaires provide valid estimates of diet measured by the Mediterranean diet score [32]. However, when diet is assessed through food frequency questionnaires, measurement error is often substantial, which could bias risk estimates toward the null [33]. The effect of measurement error is expected to increase with increasing number of measured dietary exposures [33]. This is particularly relevant in the current investigation, as several dietary factors make up the exposure of interest (Mediterranean diet score components), and are also used as adjusting covariates.

Furthermore, our results may be explained to a certain extent by residual confounding. Indeed, we found that women who adhered more closely to a Mediterranean dietary pattern were more likely to have a family history of breast cancer, to use oral contraceptives and hormone therapy, and to have a higher alcohol consumption, all of which are risk factors for breast cancer. Another concern is the single measurement of diet at baseline and the interval between diet ascertainment and breast cancer outcome. This, however, is likely to create non-differential misclassification, and would attenuate any true association.

The diet of WLH study participants is different from that of participants in studies conducted in Mediterranean countries. For example, the consumption of vegetables and fruits in this cohort was lower than in the Greek EPIC cohort, which also examined the association between Mediterranean diet and breast cancer risk [12]. While the Greek study did not report strong evidence for a beneficial effect of a Mediterranean diet on breast cancer risk (RRs were 0.88, 95% CI: 0.75–1.03; 1.01, 95% CI: 0.80–1.28; and 0.78, 95% CI: 0.62–0.98) overall, in premenopausal and postmenopausal women, respectively), it is possible that there is a consumption threshold above which beneficial components act, and that this threshold was not reached in the WLH cohort. We used a score that measured consumption patterns of different Mediterranean diet components (high or low in our study population), which should accommodate the consumption of components that might be different from other studies. However, while the consumption of more beneficial components such as vegetables and fruits was lower in the WLH cohort, the low to moderate alcohol consumption was similar to the moderate alcohol consumption in Mediterranean countries. It is therefore possible that the contribution of alcohol in higher Mediterranean diet scores was stronger in this cohort compared to studies conducted in Mediterranean countries. Our results showed a statistically significant increase in breast cancer risk in premenopausal women that was particularly strong in women with a Mediterranean diet score of 8 or 9. In our data, women with highest Mediterranean scores had the highest alcohol consumption. The increased risk might be due to residual confounding by alcohol consumption, or chance. When excluding alcohol from the Mediterranean diet score and controlled for alcohol consumption in the statistical model, the increased breast cancer risk associated with Mediterranean diet scores of 8 or 9 in premenopausal women remained.

To our knowledge, this is the first Scandinavian study that investigates the possible association between adherence to a Mediterranean dietary pattern and breast cancer risk, and no reduction in breast cancer risk was found. This was true regardless of participant characteristics, and for all breast tumor characteristics examined.
